# *DA+* data acquisition and analysis software at the Swiss Light Source macromolecular crystallography beamlines

**DOI:** 10.1107/S1600577517014503

**Published:** 2018-01-01

**Authors:** Justyna Aleksandra Wojdyla, Jakub W. Kaminski, Ezequiel Panepucci, Simon Ebner, Xiaoqiang Wang, Jose Gabadinho, Meitian Wang

**Affiliations:** a Swiss Light Source, Paul Scherrer Institute, 5232 Villigen, Switzerland

**Keywords:** protein crystallography, data acquisition software, online data analysis, beamline automation, graphical user interface

## Abstract

Data acquisition software is an essential component of modern macromolecular crystallography (MX) beamlines, enabling efficient use of beam time at synchrotron facilities. Developed at the Paul Scherrer Institute, the *DA+* data acquisition software is implemented at all three Swiss Light Source (SLS) MX beamlines. *DA+* consists of distributed services and components written in Python and Java, which communicate *via* messaging and streaming technologies. The major components of *DA+* are the user interface, acquisition engine, online processing and database. Immediate data quality feedback is achieved with distributed automatic data analysis routines. The software architecture enables exploration of the full potential of the latest instrumentation at the SLS MX beamlines, such as the SmarGon goniometer and the EIGER X 16M detector, and development of new data collection methods.

## Abstract 2

The implementation of *DA+* data acquisition and analysis software at Swiss Light Source macromolecular crystallography beamlines is presented. Detailed descriptions of the user interface, acquisition engine, online processing and database, which are the major components of *DA+*, are given.

## 1. Introduction   

Integration of hardware and software components at syn­chrotron macromolecular crystallography (MX) beamlines is essential for efficient data acquisition and online data analysis. Shorter shifts and high demand from users make it a necessity for a high-performance beamline control system, which can handle simple as well as complex data collection protocols. The control system has to be flexible enough to allow easy incorporation of new hardware and measurement protocols. At the same time, users need easy-to-use and intuitive experiment control software. In the last years, integrated graphical user interfaces (GUIs) became a standard for controlling data collection at most MX beamlines worldwide. Multiple data acquisition software and GUIs have been developed, such as *Blu-Ice* (McPhillips *et al.*, 2002 ▸), *BSS* (Ueno *et al.*, 2005 ▸), *CBASS* (Skinner *et al.*, 2006 ▸), *STARS* (Yamada *et al.*, 2008 ▸), *mxCUBE* (Gabadinho *et al.*, 2010 ▸), *JBluIce-EPICS* (Stepanov *et al.*, 2011 ▸) and *GDA* (Winter & McAuley, 2011 ▸). Sophisticated GUIs allow control of the experiment, mounting samples with robots, visualization of samples for correct alignment and, in some cases, displaying results of data analysis. Efficient use of beam time and, in turn, high productivity relies on the automatic data processing procedures, such as interfaces (González *et al.*, 2008 ▸; Incardona *et al.*, 2009 ▸; Pothineni *et al.*, 2014 ▸) and software packages (Monaco *et al.*, 2013 ▸; Winter, 2010 ▸; Vonrhein *et al.*, 2011 ▸; Tsai *et al.*, 2013 ▸). The calculation of data collection strategies allows for optimal experimental parameters resulting in higher-quality data with minimum radiation damage (Leslie *et al.*, 2002 ▸; Incardona *et al.*, 2009 ▸; Paithankar & Garman, 2010 ▸; Bourenkov & Popov, 2010 ▸; Popov & Bourenkov, 2003 ▸). Instant feedback about results during and shortly after data collection is crucial as it allows informed decisions to be made about further experiments with minimal waste of precious beam time. High levels of automation in both data acquisition and processing, coupled with the improvement of hardware, lead to the common scenario of a hundred or more datasets being collected in one user shift (8 h). The logical consequence was the introduction of a database allowing for the storage of experimental metadata and results of data processing (Pothineni *et al.*, 2014 ▸; Delagenière *et al.*, 2011 ▸). At the same time, archiving and sharing of raw X-ray data became an important factor (Meyer *et al.*, 2014 ▸; Grabowski *et al.*, 2016 ▸).

The Swiss Light Source (SLS) Macromolecular Crystallography Group operates three beamlines (X06SA, X06DA and X10SA). The software team has developed distributed *DA+* data acquisition (daq) software, which is tailored to the local setup. The design philosophy behind our daq development can be summarized in three main points: (i) intuitive and user-friendly daq protocols (minimum user instruction manual required); (ii) allows exploitation of the latest instrumentation such as multi-axis goniometer and EIGER X 16M detector; (iii) utilizes the latest technology and provides an expandable and sustainable solution supported by a small software team. In this paper we present the SLS MX data acquisition software and describe its main components. We show that our daq architecture is robust, flexible and enables exploration of the latest instrumentation such as the EIGER X 16M detector.

## 2. Hardware infrastructure   

The SLS benefits from IBM’s high-performance clustered General Parallel File System (GPFS) version 4.1 of 1.2 PB total size, of which 250 TB are dedicated for the experimental data storage for all three MX beamlines. The file server and the computing nodes are connected *via* a 40 Gbit network over an Infiniband backbone. The detector control units are connected to a file server *via* either 10 Gbit (PILATUS) or 2 × 10 Gbit (EIGER X). For online data processing each beamline is currently equipped with Dual Xeon E5-2697v2 (2.70 GHz) 24 cores, 256 GB RAM, Scientific Linux 6.4 clusters with either four (X06SA) or two (X10SA and X06DA) nodes. Three additional nodes with the same specification as the online beamline clusters are dedicated to the processing of grid scan X-ray diffraction images.

## 3. Software infrastructure   

**Figure 1:**
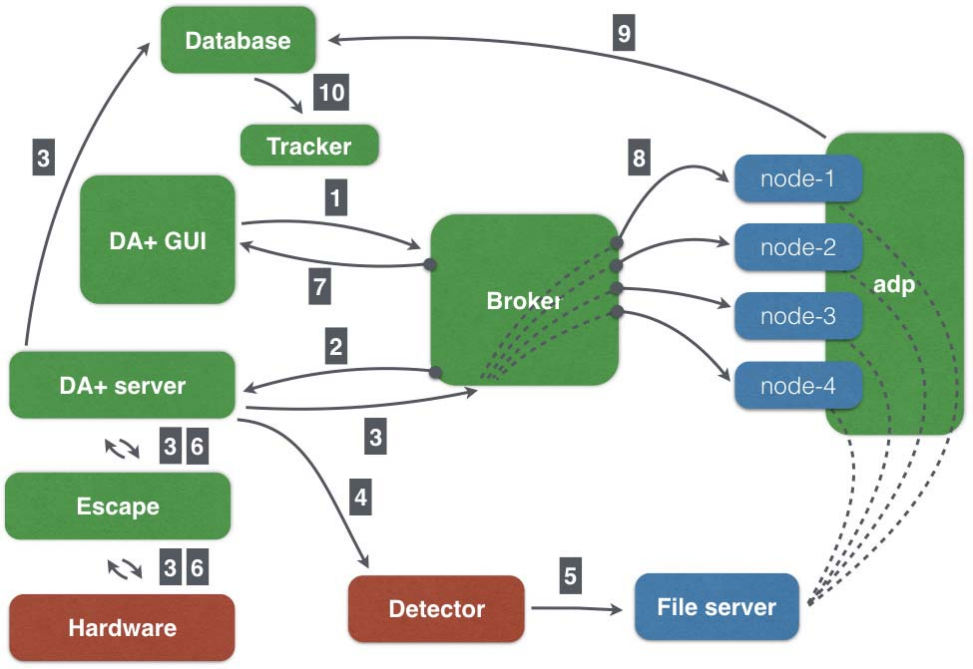
Schematic representation of the software infrastructure at the SLS MX beamlines. Software components are shown in green boxes, hardware components in red boxes, and file server and computing nodes in blue boxes. Lines indicate interactions between different components, while numbers show the order of workflow (a detailed description is given in in §3.1). The open-source message broker is a major communication hub used by *DA+* daq software components. Users control experiment parameters in the *DA+* GUI, while *DA+ server* carries out data collection and communicates with detector and hardware *via* basic state machine *escape*. *Adp* daemons receive a message from the broker, start data processing and send results to the *mxdb* database. Results of *adp* are displayed in the web-based *adp-tracker*.

The SLS MX daq system consists of distributed components written in Python 2.7 and Java (Fig. 1 ▸). Additional helper scripts used for beamline setup (such as energy change and beam position feedback) are written in Interactive Data Language (IDL). Most motors at the SLS MX beamlines are controlled through EPICS (Experimental Physics and Industrial Control System), which allows for distributed control of electronics *via* a local network. The software setup supports remote data collection at all MX beamlines. The remote access service offers access to the beamline control console in the form of a full graphical user session *via* the *NoMachine* software.

The main communication scheme implemented in our SLS MX distributed daq is *via* open-source message broker *Apache ActiveMQ* (http://activemq.apache.org/). One instance of the message broker is serving all three MX beamline. We incorporated the messaging server as a central exchange hub because it is fast, lightweight, flexible and supports multiple transport protocols. It allows for creating transparent asynchronous communication between multiple loosely coupled applications. *ActiveMQ* is versatile allowing for a publish/subscribe semantic with one message reaching many subscribers (*via* topics) and a producer/consumer semantic with one message per subscriber (*via* queues). Simple Text Orientated Messaging Protocol (STOMP), which is a simple text-based protocol similar to Hypertext Transfer Protocol (HTTP) supported by the *ActiveMQ* broker, is utilized for communication between *DA+* daq components. STOMP is language-agnostic and easily used in Python with an existing open-source client library for accessing messaging servers (https://github.com/jasonrbriggs/stomp.py). In addition to messaging *via* the broker, we utilize other communication schemes, with the Representational State Transfer (REST) being used most frequently. A lightweight alternative to Remote Procedure Calls (RPC), REST relies on stateless, client-server and cacheable communication protocols such as HTTP.

A combination of different communication styles allows for more flexible control of our resources and *DA+* daq components. Moreover, we do not rely solely on one solution and each communication protocol is tailored to the client-server requirements. REST protocols are easy to implement, straightforward to use, and provide reliable services. REST application programming interfaces (APIs) can be managed and updated without affecting other components of daq distributed software. The use of ZeroMQ streaming for EIGER X 16M data (a detailed description is given in §6) is instrumental in providing fast results of grid scan data analysis. Collecting results within one broker topic means that the number of processes handling the grid scan peak-finding task can be increased or decreased as necessary. At the same time, multiple applications are able to subscribe to the same topic and receive all the results. Depending on requirements, results can be stored or displayed (*e.g.* grid scan results in the *DA+* GUI). The messaging broker, which is the key component of our system dedicated to *DA+* server-related communication, is not overloaded and works very reliably for months without human intervention.

### 3.1. Workflow description   

The *escape* state machine is an application which is used to model experiment states and transitions between them. Distributed daq components connected *via* diverse communication schemes create a complex network of interactions, which follow a predefined path (Fig. 1 ▸). At the start of a measurement, the user aligns the sample and requests data collection in the *DA+* GUI. Subsequently, *DA+* GUI sends a message containing data collection settings to the appropriate beamline-specific broker topic (Fig. 1 ▸, step 1). The consumer of this message is *DA+ server* (Fig. 1 ▸, step 2), which communicates the request for data collection with *adp* (*automatic data processing*) background processes (daemons) *via* the broker queue and MX database *via* the REST API (Fig. 1 ▸, step 3). Subsequently, *DA+ server* issues a command to the *escape* service, which moves beamline devices *via* EPICS from the current sample alignment state to predefined positions in the data collection state (Fig. 1 ▸, step 3). In the next step, *DA+ server* sends a command to the goniometer control system, which in turn triggers the detector to collect data (Fig. 1 ▸, step 4). The resulting diffraction images are transferred from the detector control unit to the file server (Fig. 1 ▸, step 5). After data collection is finished, *DA+ server* sends a command to *escape* to transition from the data collection state back to the sample alignment state (Fig. 1 ▸, step 6) and communicates with the *DA+* GUI *via* broker (Fig. 1 ▸, step 7). Simultaneously with the execution of data collection one of the *adp* daemons receives a message with experimental details and starts data processing (Fig. 1 ▸, step 8). The results of *adp* are sent to the MX database (Fig. 1 ▸, step 9) and displayed in the *adp-tracker*, which provides user with on-the-fly feedback about the experiment (Fig. 1 ▸, step 10).

### 3.2. *Escape*   

*Escape* contains clearly defined modes for manual sample mounting, robot sample mounting, *in situ* plate operation and no movements operation. Each mode consists of multiple specific states such as sample exchange, sample alignment and data collection, which represent a defined hardware setup in time. Additional modes and states can be easily created for new measurement protocols. *Escape workflow engine* executes workflows, which define transitions to move hardware from one state to another.

The *escape* state machine is a Java application, while *escape workflow engine* is a Python 2.7 REST service. One pair of *escape* state machine and *escape workflow engine* is running per beamline. *Escape workflow engine* is active on the beamline experiment control console, while *escape* state machine is located on a dedicated remote virtual machine. *Escape workflow engine* communicates only with the *escape* state machine, which interacts with *DA+ server* *via* the REST API. *Escape* allows for asynchronous control of beamline motors movements in a predefined way and eliminates the race condition when two requests are sent at the same time. This prevents undesired behavior such as hardware collisions and undefined states. In the event of a motor malfunction the escape service goes into maintenance mode until the problem is fixed, preventing further damage.

### 3.3. *DA+* server   

Python-based *DA+ server* is the central daq component, which carries out data collection at MX beamlines. One instance of *DA+ server* per beamline is launched on the experiment control console. It initializes helper routines and scripts necessary for successful data collection and interacts with many distributed software instances, as well as hardware controllers. It also ensures that services responsible for data transfer and streaming from the detector control unit (DCU) are operational. In the case of many hardware elements, such as diffractometer stage motors, shutter, transmission filters and collimator, *DA+ server* makes sure that they are correctly positioned and in error-free status. *DA+ server* listens to messages on a beamline-specific queue in the broker and, according to the content of the message, executes or aborts data acquisition. Moreover, *DA+ server* talks to the *escape* state machine and MX database using REST APIs. It also communicates with *adp* *via* the broker.

### 3.4. *DA+* GUI   

The *DA+* GUI was developed at the SLS and is deployed at all three MX beamlines. The *DA+* GUI is implemented in Java using Eclipse RCP version 4.5 (MARS release) and is an integral part of the daq infrastructure. Apart from some beamline-specific features, the GUI is essentially identical at all three beamlines, making it straightforward to maintain and easy for users to switch between beamlines. A single instance of the *DA+* GUI is initialized on the beamline control console, from where users supervise their experiments either locally at the beamline or remotely from their institution. Modes available in the *DA+* GUI welcome window are ‘Manual mounting’, ‘Sample changer mounting’ and ‘Plate screening’ (specific to beamline X06DA). These experiment modes represent *escape* state machine modes. Plate screening enables users to test initial crystallization hits and collect data at room temperature *in situ* in an automated manner (Bingel-Erlenmeyer *et al.*, 2011 ▸). At beamline X06DA, switching to plate-screening mode including change of the robot’s gripper takes less than 2 min. During the data collection shift users can freely switch between different modes using the ‘Experiment Mode’ menu from the *DA+* GUI’s top bar.

#### 3.4.1. Data collection window   

**Figure 2:**
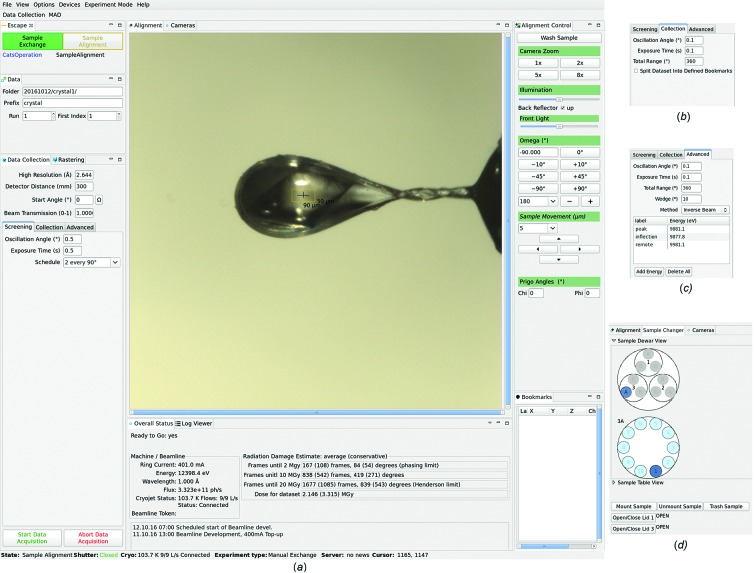
The *DA+* GUI. (*a*) The *DA+* GUI data collection window in manual mounting mode at beamline X06DA. The sample camera view is displayed in the central ‘Alignment’ tab with ‘Overall status’ and ‘Log Viewer’ tabs located below it. The ‘Escape’, ‘Data’, ‘Data Collection’, ‘Rastering’, ‘Screening’, ‘Collection’ and ‘Advanced’ tabs, which allow experimental control, are placed on the left, while the ‘Alignment Control’ and ‘Bookmarks’ tabs are on the right. (*b*) The ‘Collection’ tab with the ‘Split Dataset into Defined Bookmarks’ option. (*c*) The ‘Advanced’ data collection tab with parameters defined for *inverse_beam* MAD data collection. (*d*) The ‘Sample changer’ tab with sample dewar view. The puck in position 3A and sample in position 1 of the 3A puck are highlighted in dark blue.

Modular structure is applied throughout the *DA+* GUI to allow easy implementation of new features. The main *DA+* GUI ‘Data Collection’ window consists of multiple tabs in the ‘Manual mounting’ experiment mode (Fig. 2 ▸). The ‘Escape’ tab shows Sample Exchange and Sample Alignment modes and indicates which mode is currently active. In the ‘Data’ and ‘Data collection’ tabs the user can specify the type of experiment (screening, collection or advanced) and input experimental parameters (Fig. 2*b* ▸). The ‘Advanced’ tab allows sophisticated SAD and MAD data collection protocols to be defined, such as *inverse_beam*, *interleave_and_inverse_first*, *interleave_and_inverse_all* and *interleave_no_inverse* (Hendrickson *et al.*, 1985 ▸; Dauter, 1997 ▸; Finke *et al.*, 2016 ▸; Fig. 2*c* ▸). In the *inverse_beam* method, thin wedges of consecutive images (10–30°) are collected 180° apart. The *Interleave* method utilizes collection of thin wedges, as in *inverse_beam*, for two or more MAD wavelengths. Permutations of the *inverse_beam* and *interleave* methods give rise to *interleave_and_inverse_first*, *interleave_and_inverse_all* and *interleave_no_inverse* protocols.

At beamline X10SA the beam size can be adjusted using apertures, which can be moved in or out of the beam in the ‘Data collection’ tab. Two-stage focusing at beamline X06SA allows easy and fast change of beam size from 5 to 100 µm. The horizontal and vertical beam size can be changed with the beam focused on the sample or on the detector (default for 600 mm detector-to-sample distance). The beam size can be adjusted in an additional tab displayed in the main ‘Data collection’ window in the *DA+* GUI.

**Figure 3:**
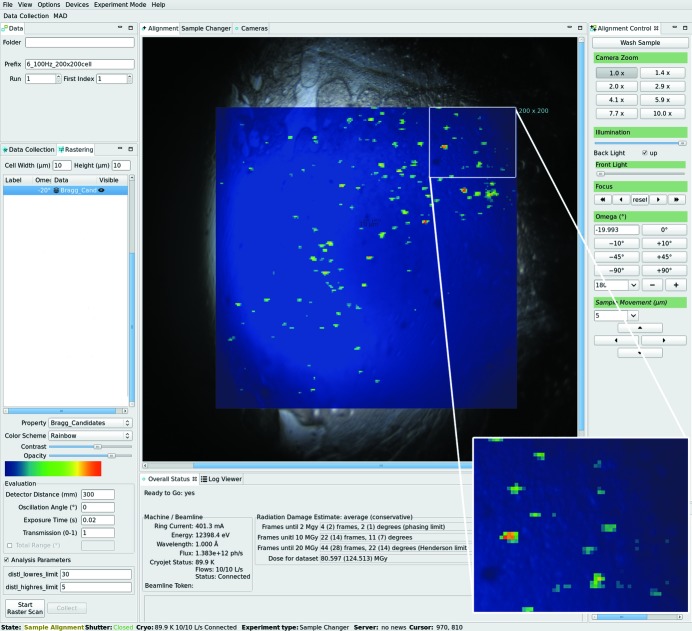
The *DA+* grid scan tab. A heat map of the diffraction results with red corresponding to the strongest diffraction is displayed on top of the sample’s camera view. A 200 × 200 cells grid scan is covering an LCP bolus containing approximately 150 membrane protein microcrystals in an *in meso in situ* serial crystallography (IMISX) plate (Huang *et al.*, 2016 ▸). Data were collected with the EIGER X 16M detector at 50 Hz with a 10 µm × 10 µm X-ray beam.

Fast continuous diffraction-based two-dimensional grid scans can be defined and executed *via* the ‘Rastering’ tab, which was described in detail previously (Wojdyla *et al.*, 2016 ▸; Fig. 3 ▸). The video image from sample camera is displayed in the ‘Alignment’ tab. Crystal centering is achieved by interactive mouse click-and-rotate procedure. The ‘Alignment control’ tab contains further parameters helpful in sample centering.

The high-precision multi-axis PRIGo goniometer (Parallel Robotics Inspired Goniometer) at beamline X06DA (Waltersperger *et al.*, 2015 ▸) allows an optimal strategy to be defined for native and experimental phasing data collection, in particular native SAD (Weinert *et al.*, 2015 ▸). PRIGo χ and φ angles can be modified in the ‘Alignment control’ tab. Future implementation of the innovative multi-axis SmarGon goniometer (a commercial version of PRIGo from SmarAct), which is characterized by compact size, high accuracy and low sphere-of-confusion, will allow continuous collection of multi-orientation data. Right-click of the mouse in the sample view allows positions to be defined within the sample for composite data collection strategies. Selected positions are shown in the sample view as magenta drops and in the ‘Bookmarks’ tab at the bottom right of the *DA+* GUI window as a list of *x*,*y*,*z* coordinates and goniometer angle. Different scenarios of data collection are available with the bookmark feature. Assuming that the user requested a 90° total oscillation range starting from ω = 0° in the ‘Data collection’ tab and defines three bookmark positions, it is possible to (i) collect the full oscillation range requested in the collection tab at each position with the same angular range, that is 90° data from ω = 0° to 90° at all three positions; (ii) split the oscillation range into each bookmark to collect discrete helical scans, *i.e.* 30° of data at each defined position with ω = 0° to 30° (position 1), 30° to 60° (position 2) and 60° to 90° (position 3). The third scenario of composite data collection is a serial crystallography (SX) protocol in which small wedges with the same angular range are collected at multiple bookmark positions. Because the SX protocol is usually utilized for samples with many microcrystals, for which only 5–10° of data can be collected, manual definition of hundreds of bookmark positions is a tedious task. Therefore, we developed an automatic software routine that identifies well diffracting crystals based on the results of a grid scan defined by the user in the *DA+* GUI and collects small wedges of data at each selected position. Incorporation of the SmarGon goniometer will allow implementation of the helical scan option into our daq software, which compliments the bookmark feature. An additional non-standard option available in the *DA+* daq software is data collection with still images. This feature can be utilized in combination with a grid scan on crystals with high mosaicity and in the case of serial X-ray crystallography data collection.

The ’Overall status’ tab displays current machine and beamline parameters such as ring current, energy, flux and cryojet temperature. It also provides radiation damage estimates based on data collection parameters. The maximum recommended number of frames for a given expected lifedose of the crystal is calculated based on the previously published equations (Holton, 2009 ▸). Average and conservative estimates are displayed for 2 MGy (the phasing limit), 10 MGy and 20 MGy (the Henderson limit) lifedoses. The ‘Log viewer’ tab displays detailed information about hardware and software controls throughout the experiment.

In the ‘Sample changer mounting’ mode an additional tab called ‘Sample Changer’ is present in the ‘Data collection’ window (between the ‘Alignment’ and ‘Cameras’ tabs; Fig. 2*d* ▸), which allows remote control of automatic sample mounting with the Cryogenic Automated Transfer System (CATS) robot (Jacquamet *et al.*, 2004 ▸). A sample trashing function, currently implemented at beamline X06DA, greatly reduces sample exchange time. Standardized spreadsheets with sample information can be loaded into the *DA+* GUI to use in combination with the CATS robot.

The *DA+* GUI ‘Data collection’ window is optimized to provide the user with all necessary tools to perform standard data collection measurements. At the same, the *DA+* GUI also allows for finer and more sophisticated control of the experiment with many optional parameters. Some parameters, such as automatic data processing options, can be changed directly in the ‘Options’ menu from the *DA+* GUI top bar. Others can be displayed in already existing tabs in the main window, for example detector height in the ‘Data collection’ tab. Additional tabs can be displayed in the main ‘Data collection’ window on demand, like the ‘Resize beam’ tab available at beamline X06SA.

#### 3.4.2. MAD window   

**Figure 4:**
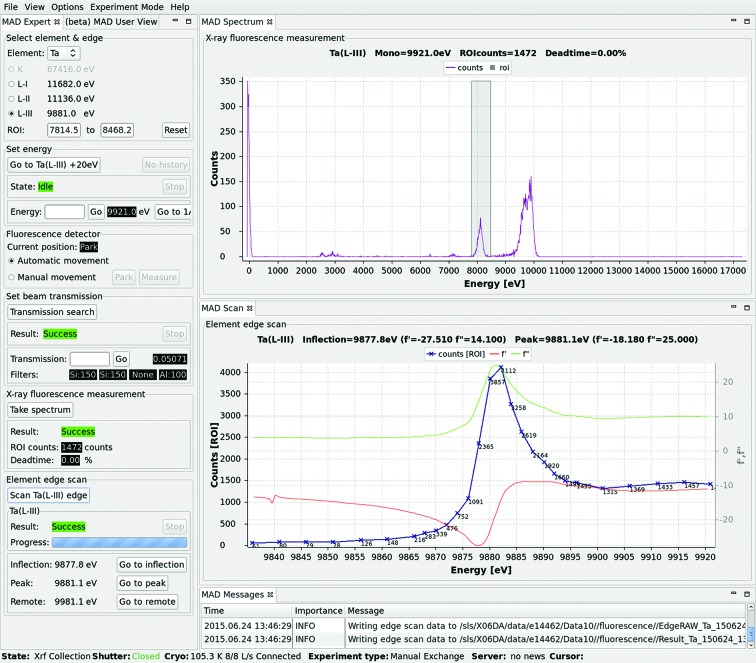
The *DA+* GUI MAD window. The ‘MAD Expert’ tab is divided into subwindows, which reflect the order of the performed steps. The ‘MAD Spectrum’ and ‘MAD Scan’ tabs plot counts against energy of a spectrum and X-ray absorption edge scan, respectively. Energies for further MAD experiments are suggested based on analysis with the program *CHOOCH*.

X-ray absorption edge scans are performed in the *DA+* GUI ‘MAD’ window (Fig. 4 ▸). The ‘MAD Expert’ tab occupies the left-hand column and is divided into subwindows, which reflect the order of steps from top to bottom. In the first subwindow, ‘Select element & edge’, the user can select an element of choice from a dropdown menu and in the ‘Select energy’ subwindow can change energy to 20 eV above the theoretical X-ray absorption edge. The same subwindow includes a button called ‘Go to 1Å’, which allows changing energy to the default native data collection wavelength. In the ‘Set beam transmission’ subwindow, the user can perform an automatic transmission search by clicking the ‘Transmission search’ button to optimize the strength of the signal. The ‘X-ray fluorescence measurement’ subwindow allows a fluorescence spectrum to be recorded with pre-defined beam transmission. The resulting raw data are plotted as number of counts *versus* energy in the ‘MAD Spectrum’ tab. The grey area labels the region of interest (ROI) for the selected element. If the number of counts for the ROI is satisfactory, scanning around the X-ray absorption edge can be performed from the ‘Element scan edge’ subwindow. The results of the edge scans are plotted in the ‘MAD Scan’ tab together with anomalous scattering factors *f*′ and *f*′′ determined using the program *CHOOCH* (Evans & Pettifer, 2001 ▸). Wavelengths for inflection, peak and high remote experiments are displayed at the bottom of the ‘MAD Expert’ tab. Energy can be changed by clicking the ‘Go to …’ button. All the raw data and results of analysis are stored in a dedicated fluorescence folder in the user’s account.

## 4. Automatic data processing   

SLS MX beamlines are equipped with three single-photon-counting hybrid pixel array detectors, namely PILATUS 6M-F (X10SA), PILATUS 2M-F (X06DA) and EIGER X 16M (X06SA). Two PILATUS detectors write data in CBF data format with one X-ray diffraction image per file (a few MB each). EIGER X 16M stores data in NeXus data format (Könnecke *et al.*, 2015 ▸) in accordance with the functional application definition for macromolecular crystallography (NXmx) with HDF5 (HDF Group, 2014 ▸) as container. Detector and experimental metadata are stored in a single master file, which contains links to single or multiple data files, each of ∼800 MB in size and containing a set of diffraction images. The *adp* routines provide near-real-time results for data in both formats. For a standard dataset with 360° total range, 0.1° oscillation and 0.1 s exposure time, fast processing of the first 180° of data is provided to the user on average 80 s before data collection is finalized. Full dataset processing, which includes multiple steps such as space group determination, resolution cutoff adjustment and conversion to *SHELX* and mtz formats, takes less than 6 min. *Adp* is written in Python 2.7 and utilizes a number of macromolecular crystallography packages, namely *XDS* (Kabsch, 2010 ▸), *POINTLESS* (Evans, 2006 ▸), *phenix.xtriage* (Zwart *et al.*, 2005 ▸), *LABELIT* (Zhang *et al.*, 2006 ▸) and *MOSFLM* (Battye *et al.*, 2011 ▸). Currently, *adp* provides a strategy based on diffraction screenshots and processing results for standard datasets. An open-source utility for managing and monitoring Unix systems called *monit* (https://mmonit.com/monit/) ensures that four *adp* daemons are active on each beamline-specific online computing cluster. The main *adp* module, called JobManager, receives a message from *DA+ server* *via* the broker queue, analyses its content, issues data processing and sends results to the MX database and the beamline-specific broker topic.

In the case of a standard dataset, *adp* is split into two steps: the ‘fast step’ (called fast_xds), which is followed by the ‘complete step’ for full processing of all data. Fast_xds is a wrapper function, which utilizes the *XDS* program package (Kabsch, 2010 ▸). To maximize the speed of data processing, fast_xds is split into three consecutive runs. The angular range chosen for each fast_xds stage depends on the total angular range of the collected dataset. For example, for a 180° dataset, fast_xds_1 is performed on 30° of data (XYCORR and INIT), fast_xds_2 on 60° (COLSPOT and IDXREF) and fast_xds_3 on 120° (DEFPIX INTEGRATE CORRECT). Fast_xds provides near-real-time feedback about the data quality, allowing the user to make a quick and educated decision about further data collection strategies. Whole data processing is performed with the in-house-developed *go.com* pipeline, which initially processes data with *XDS* in the space group *P*1 using all frames. In the next step, the space group is determined using *POINTLESS* (Evans, 2006 ▸), and data are reintegrated (if necessary) and rescaled in a new space group. In the last step, the data quality is assessed with *phenix.xtriage* (Zwart *et al.*, 2005 ▸) and final mtz file(s) are prepared. At the end of all three fast_xds stages and the *go.com* pipeline, selected output files are parsed to extract crucial statistics, which are sent to the MX database *via* the REST client.

The *adp* strategy calculation differs depending on data format. In the case of CBF format, diffraction images are indexed with *LABELIT* (Zhang *et al.*, 2006 ▸) and a strategy is calculated with *MOSFLM* (Battye *et al.*, 2011 ▸). In the case of data in HDF5 format, the initial step involves conversion to CBF data format with open-source *eiger2cbf* script (https://github.com/biochem-fan/eiger2cbf). The resulting data files contain a CBF header with correct experimental metadata that allow indexing and strategy calculations to be performed with *MOSFLM*.

## 5. Database and tracker   

**Figure 5:**
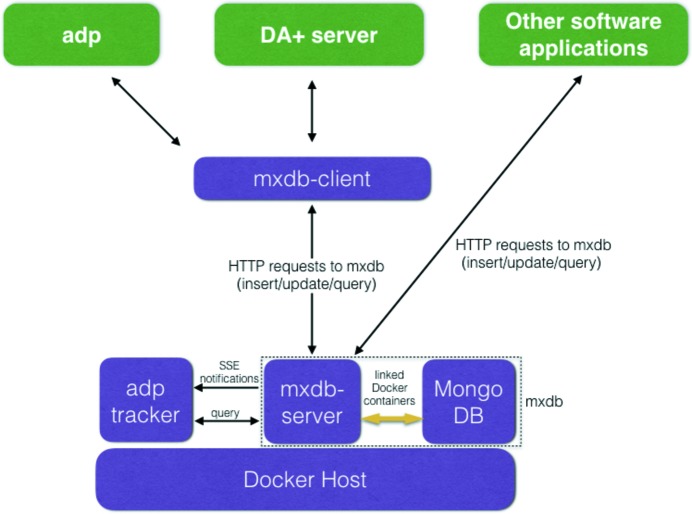
Schematic of communication with *mxdb*. *Mxdb-server*, *adp-tracker* and *MongoDB* are deployed as Docker containers. *Mxdb-server* and *MongoDB* containers are linked, with *mxdb-server* providing the only access point to *MongoDB*. Python applications such as *adp* and *DA+ server* communicate with *mxdb-server* *via* *mxdb-client*. Non-Pythonic applications running at the beamline communicate with *mxdb-server* directly through HTTP requests. *Adp-tracker* relies on Server Side Events (SSEs) emitted by *mxdb-server* to query *mxdb* and update its display.

An *mxdb* database system, which receives and stores all the metadata produced by users and beamline equipment during measurements, serves all three SLS MX beamlines. Primarily, the database collects the information related to the experiment conditions (for example X-ray energy, beam size and location of the diffraction images), as well as results of the *adp*. Additionally, it tracks operational parameters of hardware units critical for beamline operation and accuracy of measurements. The main purpose of *mxdb* is to store the information for the post-beam time analysis by users and to provide staff with statistical information about beamlines usage patterns. *Mxdb* consist of the database engine and the web server (*mxdb-server*), which allows communication with external services (Fig. 5 ▸). The *mxdb-server* is a Python 2.7 application written with the Flask microframework. It provides the RESTful API to insert/update documents to the database and to retrieve them with query syntax mimicking *MongoDB* query types (*i.e.* *find*, *findOne*, *distinct*, *aggregate*). The API accepts and returns messages in universal JSON format. This significantly simplifies accessing *mxdb* from other applications running at the beamline, irrespective of the programming language, as, instead of installing, implementing and maintaining additional language-specific *MongoDB* drivers, all they need to provide is an HTTP request.

We benefit from a *MongoDB* (https://www.mongodb.com/) schemaless design, which does not enforce strict format of the data storage. This enables us to easily adapt *mxdb* to new kinds of experiment protocols and reorganize existing data to facilitate its display and analysis by other applications, for instance *adp-tracker*. We enforce a simple level of storage organization, which keeps datasets in a tree-like structure (‘project/target/crystal’). Prior to the insertion to the database, each message is parsed at the *mxdb-server* to assure its correct insert point or reference to the location in the tree structure. The parser additionally checks data consistency; for instance, ensures identical labels for each beamline name, formats timestamps or checks for the existence of predefined fields that have to be present in every document in the database. Both components of *mxdb*, *i.e.* *MongoDB* and *mxdb-server*, are deployed using linked Docker containers, where *mxdb-server* is the only access point to the *MongoDB* instance. To ease the communication and reduce the boilerplate code for other applications that require access to *mxdb* we have created the *mxdb-client* module, which wraps the most commonly used *mxdb-server* REST calls in a convenient-to-use Python class (Fig. 5 ▸).

**Figure 6:**
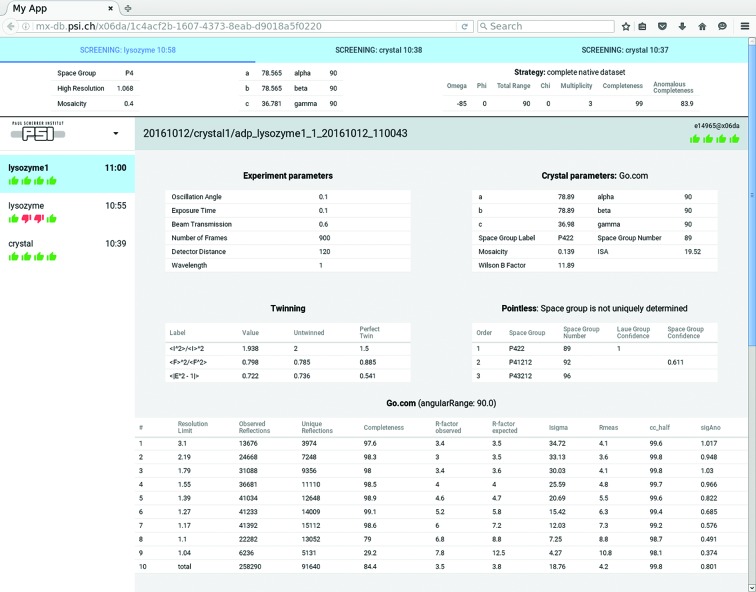
Results of *adp* shown in the web-based *adp-tracker*. The top window displays results of strategy calculation for the last screenshots. Below, results from the last step of automatic data processing with the in-house *go.com* pipeline for a sample called lyzosyme1 are displayed. Tables include experiment parameters, crystal parameters, twinning analysis, *POINTLESS* space group determination and final xds CORRECT.LP table.

Apart from storing data for further retrieval and analysis, the *mxdb* serves as a backbone for online services available at the beamline during user measurement shifts. It implements a notification system based on Server Sides Events (SSE), which streams documents arriving to selected collections in the database to subscribed client applications. Currently the main consumer is *adp-tracker*, which provides real-time display of *adp* jobs as they progress with data analysis during user beam time. The *adp-tracker* is a web application written utilizing HTML5 Web Components standard based on Google Polymer Library (https://www.polymer-project.org/). Currently it is accessed *via* a web browser from the beamline control console to provide the user with real-time feedback about the quality of the ongoing data collection. In the future both *mxdb* and *adp-tracker* will be accessible to remote users. The design of *adp-tracker* was deliberately chosen to be compatible with mobile and tablet devices. The left-hand panel of *adp-tracker* application available during measurements at the beamline shows a sorted list with collected datasets (Fig. 6 ▸). Each element of this list indicates the name of the dataset, the time when the data collection was started, and the status of different stages of automated data processing: ‘Initialization’, ‘Indexing’, ‘Fast Processing’ and ‘Go.com’ are symbolized by icons below the dataset name. Each notification, arriving as the SSE from *mxdb*, triggers an automatic update of the information in a list of datasets. This could be either the start of new dataset processing (appearing on the top of the list) or a change of the status of one of the *adp* steps. The available statuses are ‘pending’, ‘running’, ‘success’, ‘failed’ and ‘canceled’, and each one is symbolized by a different icon. On the right of the dataset list, *adp-tracker* shows the main window where detailed information on a selected dataset is presented. It is presented in tables, where each table shows the result from one *adp* processing stage. Whenever an *adp* stage is finished, a new table or error message (for example, due to bad data quality) appears in the view window of the currently processed dataset. To keep *adp-tracker* uncluttered and explicit we display the results of only the five latest strategy calculations in the top vertical bar above the main window (Fig. 6 ▸).

## 6. EIGER implementation   

**Figure 7:**
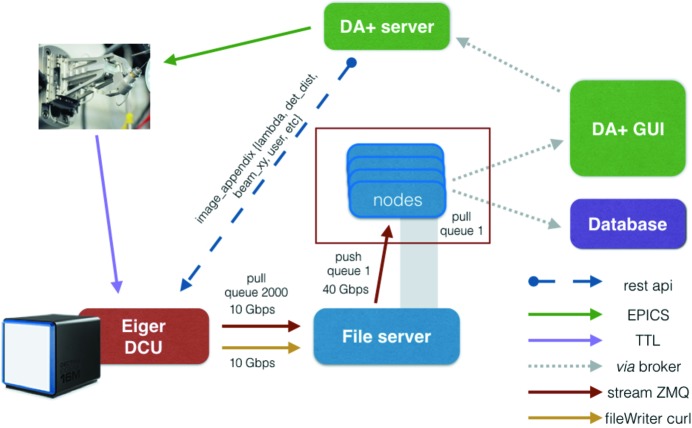
Schematics of the communication network required for EIGER X 16M operation at beamline X06SA. *FileWriter* is used for writing data files to GPFS and for displaying diffraction images in the ALBULA diffraction viewer. Streaming of the grid scan diffraction images to the memory of the computing nodes allows online analysis. *DA+ server* sends *image_appendix*, which contains all experimental metadata required for processing of diffraction images, such as detector distance, wavelength and beam position.

One of the recent major hardware upgrades was installation of the first Dectris EIGER X 16M detector at beamline X06SA. Because of the flexibility of the MX SLS daq software, integration of the new detector into our infrastructure proved straightforward. The RESTlike SIMPLON API (Dectris) provides platform-independent access to the EIGER X DCU. In-house-developed Python-based software allows communication with DCU *via* dedicated clients. The full potential of the EIGER X 16M detector and maximum speed of data processing were achieved by utilization of multiple communication schemes (Fig. 7 ▸). After ensuring that beamline hardware is in the correct state and the detector configured according to user-defined data collection parameters, *DA+ server* communicates with the goniometer control system (Aerotech or SmarGon) *via* EPICS (Fig. 7 ▸, green line). The Aerotech controller, which drives the data acquisition stages (Ω and/or *X* or *Y*) issues a position synchronized TTL signal, which opens the shutter and triggers the detector (Fig. 7 ▸, magenta line). The fileWriter, which writes metadata and frames to the HDF5 files, is started. Resulting data files are transferred from the DCU to the file server *via* one of the 10 Gb connections with cURL using the Web Distributed Authorizing and Versioning (WebDAV) protocol (Fig. 7 ▸, yellow line). Data stored on the file server are displayed in the ALBULA diffraction viewer (Dectris) on the user console and processed with *adp* in the case of standard data collection. Results of *adp* are delivered to the *mxdb* database *via* the broker (Fig. 7 ▸, gray dotted line). In the case of grid scan data collection, *DA+ server* activates not only the fileWriter but also the stream module. *DA+ server* sends image_appendix, which contains all experimental metadata required for processing of diffraction images (Fig. 7 ▸, blue dash line). Image and header data are transferred *via* ZeroMQ sockets in the Push/Pull scheme (http://zeromq.org/) using the second 10 Gb connection between the DCU and file server. Each ‘Image data’ ZeroMQ multipart message contains the bit-shuffled (32 bit) and lz4-compressed data (https://github.com/kiyo-masui/bitshuffle) and the image_appendix. The SIMPLON API server opens the ZeroMQ Push socket and a dedicated Python-based client on the file server (called *mflow_splitter*) opens the ZeroMQ Pull socket with a queue size of 2000 messages (Fig. 7 ▸, red line). The *mflow_splitter* client forwards the incoming ZeroMQ messages further; it generates streams and pushes messages (queue size of 1 message) to grid scan daemons on the computing nodes *via* the 40 Gb Infiniband connection (Fig. 7 ▸, red line). There are 140 dedicated grid scan daemon processes monitored by the *monit* utility and distributed over seven online computing nodes, which consume incoming messages. Daemons decompress the data and process it using *labelit.distl* package routines (Zhang *et al.*, 2006 ▸) or *Cheetah*’s *peakfinder8* routine (Barty *et al.*, 2014 ▸), while in the memory of the computing nodes. Results are reported back to the *DA+* GUI *via* the broker (Fig. 7 ▸, gray dotted line).

EIGER X 16M can achieve frame rates of 133 Hz for full frame; however, the ROI feature enables a reduced area of the detector to be read out with increased speed. The 4 M ROI readout of EIGER X 16M, which covers eight central modules, allows a frame rate of 750 Hz to be reached. Users can configure the ROI in the *DA+* GUI for both standard data collection (default is 16 M) and grid scan (default is 4 M). The 4 M ROI is particularly suitable for fast grid scans on large samples with multiple microcrystals, such as solid supports (Hunter *et al.*, 2014 ▸; Feld *et al.*, 2015 ▸) and *in meso in situ* serial crystallography (IMISX) plates (Huang *et al.*, 2015 ▸, 2016 ▸) as the 4 M images are evaluated in a fraction of the time needed for a 16 M image. An example of a grid scan performed on a microcrystal with the microbeam at 50 Hz is shown in Fig. 3 ▸. The planned upgrade of the SLS storage ring and, consequently, the increase in the beam flux density makes grid scanning at 500 Hz within our reach. It is crucial for the innovative hardware solutions to be matched by computer power. We are, therefore, in the process of upgrading the X06SA online computing nodes to ensure on-the-fly data processing of data collected at such high frame rates.

## 7. Summary   

In-house-developed distributed *DA+* daq software has been implemented at all three SLS MX beamlines. It benefits from versatile communication schemes, with messaging and REST APIs being the two main modes. *DA+* daq provides an easy and intuitive GUI, which allows straightforward experiment control. The *DA+* GUI supports both simple and complex data collection strategies and provides different levels of experiment parameters control, such as on-demand tailored beam size, detector height offset or expert tab for beamline setup (available to beamline staff). Fast online automatic data processing provides users with data collection strategies and instant feedback about data quality, which are displayed in the *adp-tracker*. Experiment metadata and *adp* results are stored in the *MongoDB* database. In the future, *adp* routines will be expanded to cover a wider range of data collection protocols, in particular merging for *inverse_beam*, *energy_interleave*, native-SAD and serial crystallography experiments. This will be complemented by the user interface, which allows remote browsing of data stored in the database and scheduling of data processing jobs. Further improvements include the option of fully automatic sample screening based on information provided in the spreadsheet file loaded into the *DA+* GUI prior to the experiment. The installation of an EIGER X 16M at the X06SA beamline, which can achieve frame rates of 133 Hz for the full frame and 750 Hz for 4 M ROI, significantly increased crystal screening and data collection speed. Optimal management of computing resources, combined with efficient communication and software solutions, provides users with data processing results in near real time in the case of both standard data collection and fast grid scan.

The multicomponent SLS MX *DA+* daq is fully functional, and yet flexible enough to adapt to an always-evolving beamline environment, whether it is new hardware, software or a data collection method. Overall we are using state-of-the-art hardware and software solutions, which keep SLS MX beamlines in the forefront of the current landscape of the synchrotron MX world, and form a solid foundation for further development in the foreseen diffraction-limited SLS storage ring upgrade.
